# Facts and Cases, Reported for the Western Journal

**Published:** 1832

**Authors:** William Maclay Awl

**Affiliations:** Somerset, Ohio


ART. II.—*Facts and Cases, reported for the Western Journal.—By Wm. Maclay Awl, of Somerset Ohio.*

A country practitioner in the West, most frequently has his location, as it were, upon the confines of the learned world, where both the labors of his profession, and the common concerns of life unite, to unfit, and disincline his mind for any thing like speculative disquisition, or original communication. His necessity obliges him to consult only with *facts*, in relation to the emergencies of his profession, and generally without the intervention of much theory upon the subject.

But as *facts* and *cases*, may be denominated the raw materials of the profession capable only of the finer finish of the market, and you appear to possess an honest claim upon the interior for the commodity on hand, I propose occasionally to gather in a brief style, some of the lessons experience may teach, and in the shape of private clinical reports, transmit them for the pages of your journal.

*Paracenthesis Thoracis.* My practice affords another instance of the success which may attend this operation, as the following case will testify. Joseph Woods, aged 28 years, of stout make and good constitution, was attacked on the first of May 1830, while engaged at work upon the Ohio canal, with ordinary pleuritis. A neighboring physician bled him once triflingly, put a small blister upon the affected side, and gave various medicines, from all of which he obtained no permanent benefit, but continued to linger up and down in great misery, with an unsubdued and fixed disease, in mighty conflict against his stronger nature. Suddenly he felt something crack and give way within his breast, like the report of "a common pop-gun." He sprang up, and the matter gushed from both nose and mouth in a full stream; it continued to run freely, until more than two quarts were discharged, when it gradually ceased, leaving him much relieved of the pain and distress, under which he had then suffered for 3 weeks. Thereafter he made out to hobble home, a distance of 25 miles, and there continued sick for a long time, the matter every now and then gushing up into his throat, and passing out in large quantities. Sometimes it would come up so suddenly, particularly if he were in a recumbent posture, as to threaten instant strangulation, and if occasionally he slept, it was most distressing to witness the heavy anguish of his chest, while the pus was frequently thrown into the trachea with such force, that it escaped by either nose, or mouth, as they became the most defending parts.

Sometime after this a physician was consulted, who pronounced him in the middle of a liver disease—and promising a very speedy cure, he commenced, to employ his own appropriate language, "*to run the marcury as close to him as he dare, without injuring of him,*"—taking especial care at the same time, to wash down the doses, with liverworst tea, that plant being then, just in the zenith of its popular fame. Under this administration he was suffered to continue for three or four months, growing worse and more weak every day, and unable for the last ten weeks to lie down at all. His suffocating cough was almost incessant, and its daily increase presenting nothing but the horrid prospect of sudden strangulation, he one day sent for his Doctor, and expressed a wish to be cut open, believing that if that could be done, he would be cured.

Under these circumstances I was sent for, at the distance of 12 miles, to consult about the propriety of such an operation, and for the first time saw the patient. With his thin elbows braced out upon pillows, he sat before a small table, the perfect picture of despair—there had been no chills, or symptoms of fever since the pus began to be discharged. On the contrary his skin was pale and cool; no particular thirst or bad feelings about the stomach, and so far from any thing like a liver affection, his tongue was perfectly natural, the bowels in good condition, and the whole digestive system in excellent health—his appetite might indeed be called good, though what was eaten, proved of little service to him, for the violent fits of coughing, most commonly ended in vomiting, and then the food was always discharged. But he had not been able to lie down for several weeks, and his legs had become œdematous to the knees. Upon examination, there appeared an evident fulness under the lower ribs of his right side. I measured round each side from the point of the ensiform cartillage to the spine, and a careful comparison made the affected side half an inch the largest. Next I tried percussion with the point of my finger, sounding all round in every direction, until, with indubitable satisfaction, I determined the centre of the tumor to be between the middle of the 6th and 7th ribs of the patient's right side. The integuments were divided in the usual manner, and a flat trocar passed through the pleura. Three quarts of pus were discharged, and then the air whizzed clear. It was truly astonishing to see, how to the last drop the lung would punp it out. By passing in a small probe, after it had ceased running, I determined that the direct depth of this vast abscess exceeded 5 inches—more than half way through the body of the right lung! The poor fellow was instantly and perfectly relieved, and immediately laid down, rejoicing in the satisfaction of once more resting quietly in his bed. It was my intention to have the wound kept constantly open, and I accordingly had a small canula constructed expressly for the purpose, but under every contrivance it caused so much irritation and cough, that it could not be continued, and very much to my disappointment, I had at the expiration of ten days, to go and perform the operation over again, when nearly a similar quantity of pus was again discharged. To prevent the like occurrence in future, I instructed his mother to introduce the blunt end of a female cathetre twice a day, and evacuate each time, any matter that might exist. This arrangement answered much better, and he very soon began to improve. The swelling quit the extremities without any particular remedy, in a few days after, he became able to lie down, and the cough and irritation gradually abated.

The discharge from the wound was less and less, until in six or eight months it entirely dried up, and the wound closed over, and finally, without the help of any other medicine than one or two Dover powders, he continued rapidly to improve until his health became perfectly and fully established; and he remains at this time strong and firm.

*Chloride of Lime in Ozena.*—I take great pleasure in being able by the following case, to corroborate the success of this valuable remedy in chronic purulent discharge from the nose, recommended in the XI. No. of "The American Journal of the Medical Sciences," by William E. Horner, M. D., adjunct professor of Anatomy in the University of Pennsylvania. It is a most intolerable and filthy disorder which has hitherto, I believe, resisted the ingenuity of the most respectable in surgical skill, and the prospect of a certain remedy will doubtless claim for its discoverer the thanks and respect of the Profession.

Early last spring I was requested to prescribe for this offensive disorder, by Mr. H——, a most respectable and worthy citizen of Somerset, Ohio. He was by profession a chair maker and sign painter, and 37 years of age. With the exception of painters' cholic, to which he was occasionally subject, his constitution and health might be called good. He could assign no particular cause for the disorder, unless it had been excited by the frequent colds to which he had been subject in the preceding fall, or that the lead had effected his nose also. His character was a sufficient guarrantee against any suspicion of syphilis. The disease had first begun about the commencement of January, 1831, by repeated and severe attacks of acute pain above the eye-brows and lower parts of the forehead, to which was often added, when the sufferings became most severe, more or less pain and inflammation in the eyes themselves. Bleeding, purging, blistering, and various other remedies had been used with little or no benefit, and the attacks continued to increase in frequency and severity for two months, when the nose suddenly opened, and there followed a copious discharge of the most offensive muco-purulent matter; it was from but the one nostril at first, though the other subsequently became affected. The running was most troublesome and abundant early in the morning, the nostril by that time having become perfectly full. At night, especially, if he chanced to sleep upon the back, the matter would run down his throat, and by its offensive character produce so much sickness as most generally to destroy all appetite, at least, for breakfast. During the day, the ready opportunity of blowing, rendered the discharge much less disagreeable, but the constant effort kept his nose always uneasy, and more or less painfully inflamed, and if any sudden cold, to which he appeared remarkably subject, caused the running to stop, the original pain and distress returned immediately to the forehead, nor would it again be relieved, until a more copious discharge ensued.

At my request he commenced the use of the chloride of lime on the first of March, 1831, by putting a tea-spoonful into a cup of water, and injecting the clear liquor three times a day, high up into the nostril. Its effects were at first very severe, made him sneeze terribly, and he did not continue it long before it produced both so much pain and hæmorrhage, as obliged him for a week to suspend it altogether. At the end of that time he began again, the effect was not so severe as before, and he determined to persevere. It always produced a more copious discharge, and did much service in correcting the fetor of the matter, but he had continued it three times every day for at least four weeks before he was satisfied that it was producing any permanent change. Nearly about the same time, the other nostril also commenced running, after which he improved so fast, that by the end of June the cure was complete. It has not since returned in the slightest degree.

*Premature Labor.*—May the 1st, 1830, Mrs. D. was taken suddenly with pains, resembling labor; she was in the seventh month of her pregnancy. The pains had immediately supervened upon the accidental rupture of the membranes and the discharge of the liquor amnii. It was the first conception, and on that account and her small size, any satisfactory examination was very difficult. I was satisfied, however, that the membranes had actually given way, and that the discharge came really from within the uterus, for it was quite copious, and would gush forth most especially in company with the regular pains, but the uterus had evidently been taken entirely by surprise, as there was not the least apparent disposition on the part of nature to prepare for an actual labor. Venesection was performed; the bowels emptied with a spoonful of castor oil; and instructions given to remain as quiet as possible. As much opium was given from time to time as the urgency of the pains demanded. I expected that labor would ultimately be produced, and was curious to watch the time, at which under the circumstances it would commence, all things remaining undisturbed. It was thirteen days before it actually ensued, and then it was conducted in the most lingering, and on the part of nature, apparently most unwilling manner. It proved to be a footling case, and one leg having escaped through the rent in the membrane, could be felt moving strongly within the vagina. Eventually, the delivery was accomplished with safety to both mother and infant, the latter being snug in the membranes, which were unbroken, except the little hole in the bottom, from which the leg protruded. Dr. Dewees ask, why it happens, that in the majority of premature labors, the cases are footlings? In the instance just detailed, the fact is explained by the manner in which the membranes were ruptured by the leg of the child.

